# Supplementary figures and images for: Distribution and Genomic Characterization of Third-Generation Cephalosporin-Resistant Escherichia coli Isolated from a Single Family and Home Environment: A 2-Year Longitudinal Study
Source: Antibiotics (Basel). 2022 Aug 25;11(9):1152. doi: 10.3390/antibiotics11091152 (PMC9495048; doi:10.3390/antibiotics11091152)

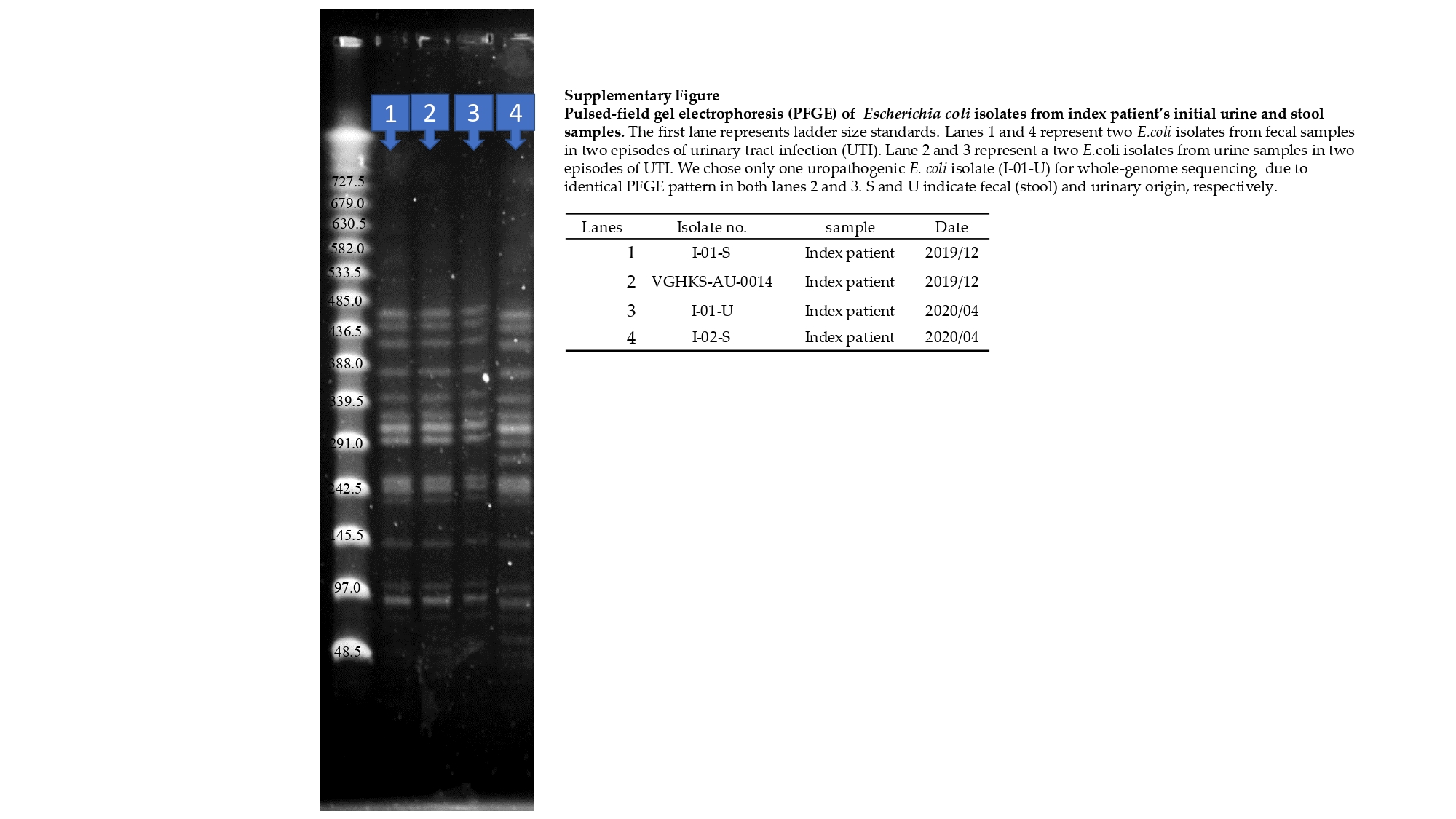

Supplement: Supplementary file 1 [file antibiotics-11-01152-s001.zip › Supplementary Figure.jpg]

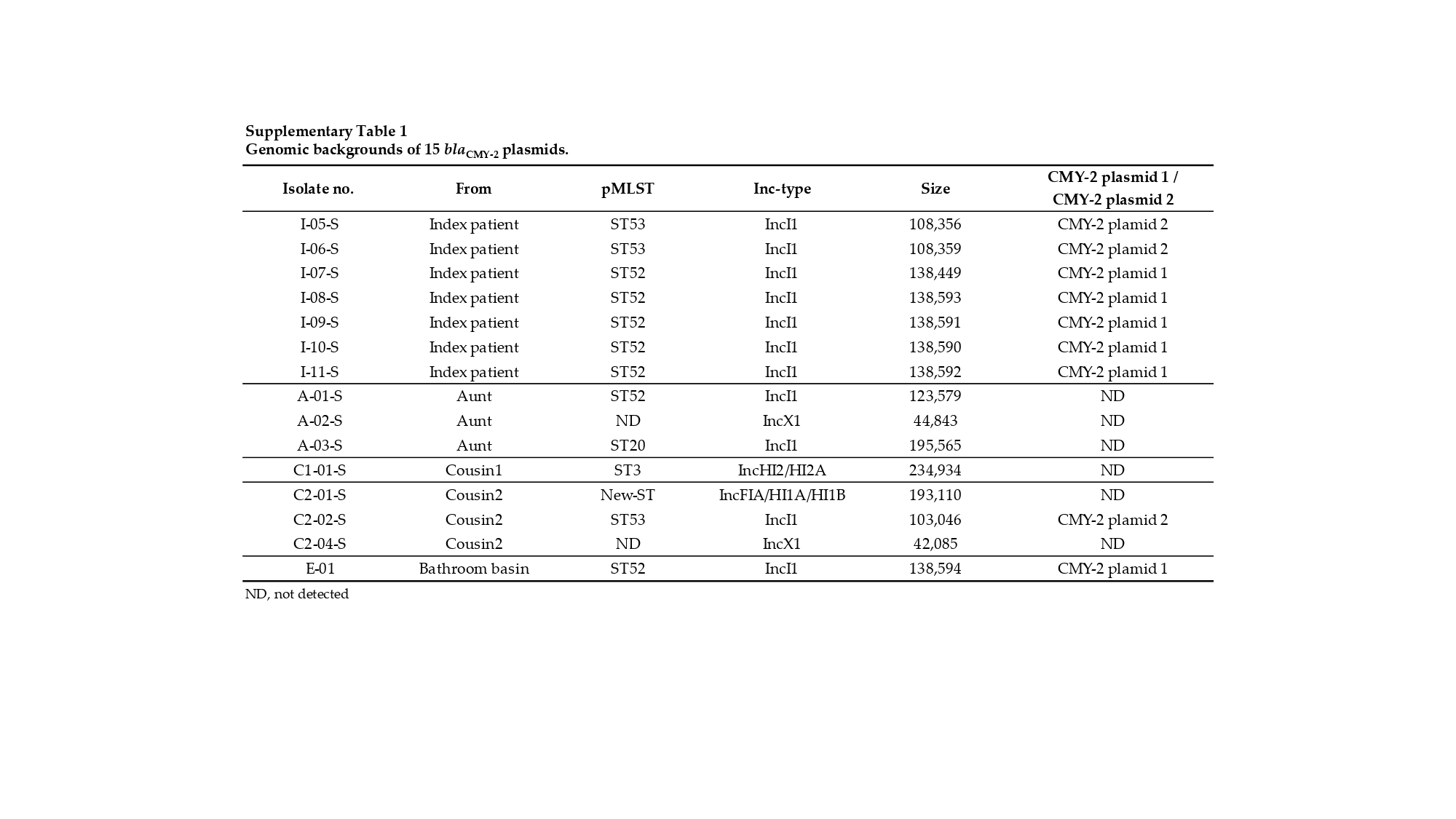

Supplement: Supplementary file 1 [file antibiotics-11-01152-s001.zip › Supplementary Table S1.jpg]

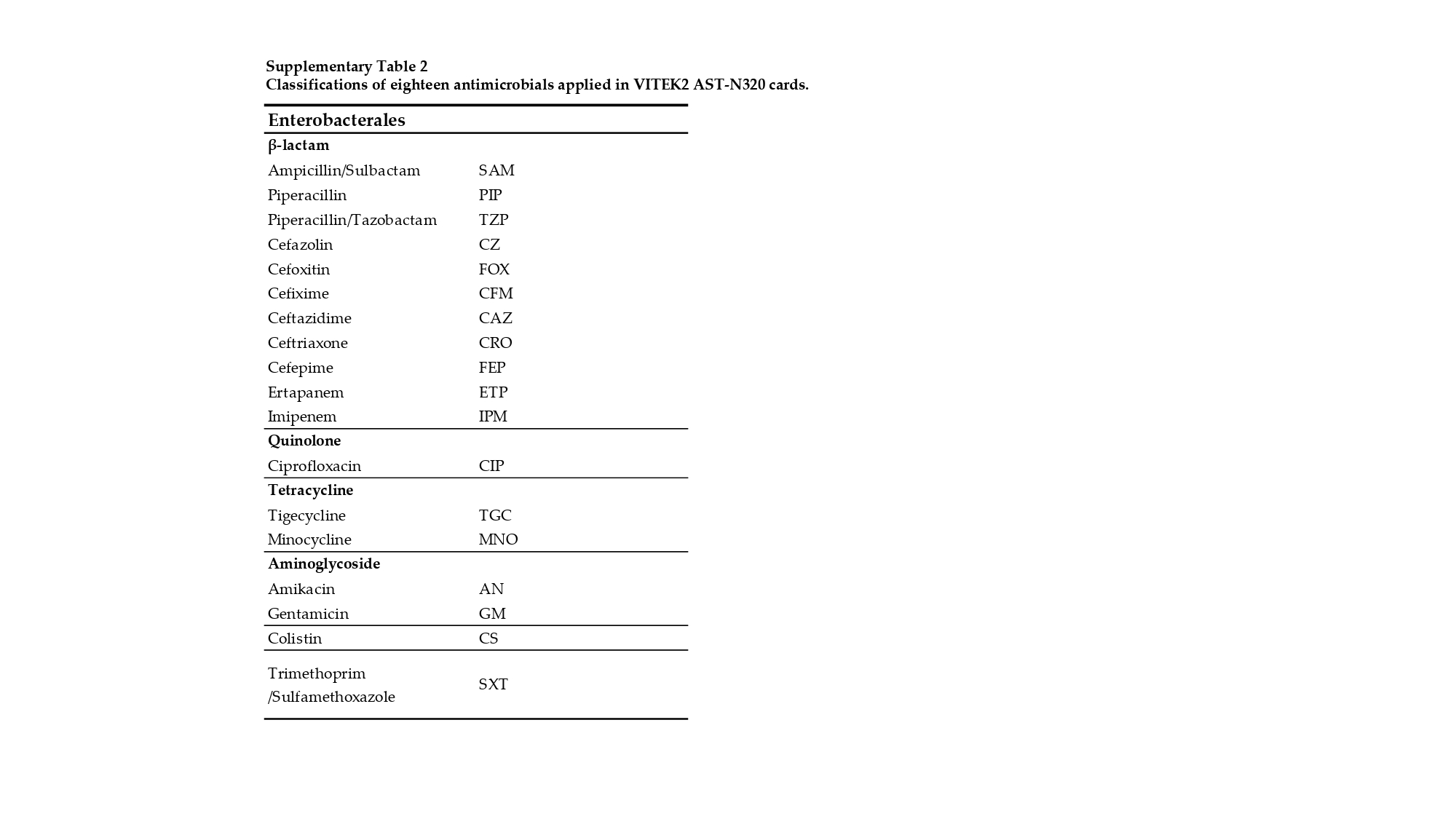

Supplement: Supplementary file 1 [file antibiotics-11-01152-s001.zip › Supplementary Table S2.jpg]
